# Pharmacological Properties of Riparin IV in Models of Pain and Inflammation

**DOI:** 10.3390/molecules21121757

**Published:** 2016-12-21

**Authors:** Olívia Azevêdo Nascimento, Renan Fernandes do Espírito-Santo, Luíza Carolina França Opretzka, José Maria Barbosa-Filho, Stanley Juan Chavez Gutierrez, Cristiane Flora Villarreal, Milena Botelho Pereira Soares

**Affiliations:** 1 Faculdade de Farmácia, Universidade Federal da Bahia, 40170-290 Salvador, BA, Brazil; olivia_azevedo@hotmail.com (O.A.N.); luizacfo@ufba.br (L.C.F.O.); 2 Centro de Pesquisas Gonçalo Moniz, FIOCRUZ, 40296-710 Salvador, BA, Brazil; r.fernandes88@hotmail.com (R.F.d.E.-S.); milena@bahia.fiocruz.br (M.B.P.S.); 3 Laboratório de Tecnologia Farmacêutica, Universidade Federal da Paraíba, 58051-900 João Pessoa, PB, Brazil; barbosa.ufpb@gmail.com; 4 Departamento de Bioquímica e Farmacologia, Universidade Federal do Piauí, 64049-550 Teresina, PI, Brazil; stanleychavez@ufpi.edu.br; 5 Centro de Biotecnologia e Terapia Celular, Hospital São Rafael, 41253-190 Salvador, BA, Brazil

**Keywords:** anti-inflammatory, antinociception, PGE_2_, cytokines, riparins

## Abstract

Riparins, natural alkaloids of the alkamide group, can be synthesized by simple methods, enhancing their potential application in pharmaceutical development. Here, the pharmacological properties of riparins were investigated in in vitro and in vivo assays of pain and inflammation in Swiss mice. Inflammatory mediators were measured by radioimmunoassay and Real-Time PCR. Riparins I, II, III and IV (1.56–100 mg/kg; ip) produced dose-related antinociceptive effects in the formalin test, exhibiting ED_50_ values of 22.93, 114.2, 31.05 and 6.63 mg/kg, respectively. Taking the greater potency as steering parameter, riparin IV was further investigated. Riparin IV did not produce antinociceptive effect on the tail flick, suggesting that its antinociception is not a centrally-mediated action. In fact, riparin IV (1.56–25 mg/kg) produced dose-related antinociceptive and antiedematogenic effects on the complete Freund’s adjuvant (CFA)-induced paw inflammation in mice. During CFA-induced inflammation, riparin IV did not modulate either the production of cytokines, TNF-α and IL-10, or COX-2 mRNA expression. On the other hand, riparin IV decreased the PGE_2_ levels in the inflamed paw. In in vitro assays, riparin IV did not exhibit suppressive activities in activated macrophages. These results indicate, for the first time, that riparin IV induces antinociceptive and anti-inflammatory effects, possibly through the inhibition of prostanoid production.

## 1. Introduction

Inflammation is a body’s protective response to infection, tissue injury and other noxious conditions. On the other hand, the outcome may be deleterious if it leads to chronic inflammation, which stimulates the development of many diseases, such as atherosclerosis, diabetes, neurodegenerative diseases and cancer [1]. Therefore, the appropriate treatment for inflammatory disorders must be based on a therapeutic capable of gaining rapid control the inflammation, preventing tissue damage and achieving disease remission. These goals can hardly are achieved by the pharmacological treatments currently available, stressing the importance of developing new drugs.

The clinical treatment of inflammatory diseases is dependent on non-steroidal or steroidal chemical drugs [2]. Glucocorticoids remain the most effective treatment of inflammatory and immune diseases. On the other hand, many patients exhibit resistance and severe side effects to glucocorticoids, mainly during prolonged treatments [3,4]. Nonsteroidal anti-inflammatory drugs (NSAIDs) have also been extensively used worldwide for both chronic and acute musculoskeletal and inflammatory conditions. However, NSAIDs are associated with several side effects such as gastro-intestinal ulcers, bleeding, renal disorders, cardiovascular events and hypersensitivity reactions [5,6].

A large number of studies have been performed in an attempt to identify alternative drugs from natural sources that effectively interact with the immune system without causing adverse effects. Plant secondary metabolites are important for flavoring of food, resistance against pests, as well as drugs with a variety of pharmacological properties, including anti-inflammatory activity [7,8,9,10,11]. From the data presented in a recent review by Newman and Cragg, natural products are an important source for discovery and development of the final drug entity today [12]. However, natural product-based drug discovery is associated with some intrinsic difficulties, such as low amount of natural substances available and seasonal variability, which generally makes their commercial exploitation unfeasible [13]. Therefore, efforts have been made to produce natural substances and their derivatives by chemical synthesis. This approach allows the pharmacophore to be recognized, as well as the optimization of pharmacological properties, enhancing the potential of natural substances to pharmaceutical development [12,13].

Some alkaloids of the alkamide group were isolated from the green fruit of *Aniba riparia* (Nees) Mez, a Lauraceae plant endemic of the Amazon forest (Brazil) popularly known as “louro” [14]. Its natural alkaloids, known as riparins, showed antimicrobial, anxiolytic, antinociceptive and anti-inflammatory activities in preclinical studies [14,15,16,17,18]. These properties awakened interest in verifying the pharmacological potential of these amides, which can be obtained synthetically by condensation between acyl chlorides and O-methyltyramine in a yield of until 93% [19]. In addition, several structural analogues of riparins have been synthesized from simple methods, increasing the pharmaceutical potential of these molecules. The present study was undertaken to establish the pharmacological properties of riparin IV in in vitro and in vivo assays of pain and inflammation. We described here, for the first time, that riparin IV possesses antinociceptive and anti-inflammatory effects deprived of immunosuppressive action, possibly related to its ability to inhibit the production of PGE_2_. 

## 2. Results and Discussion

**Figure 1:**
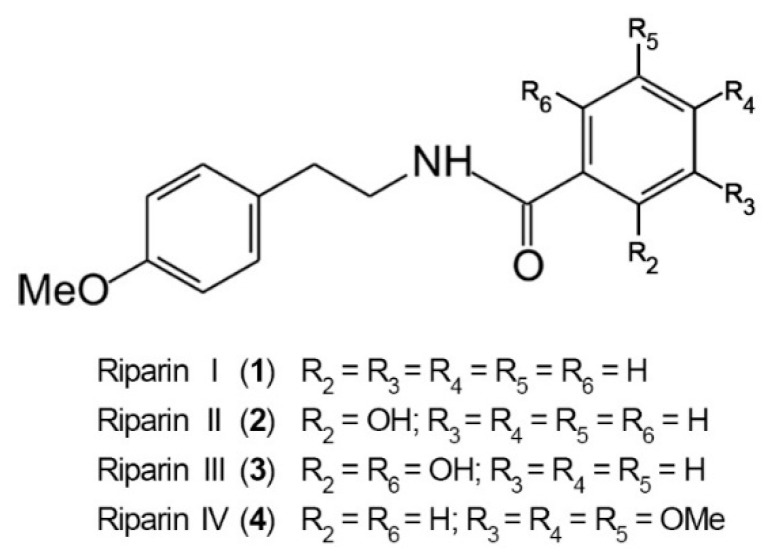
Chemical structure of riparins I, II, III and IV.

The antinociceptive properties of riparins were initially evaluated using the formalin test in mice, a screening tool for the assessment of analgesic or anti-inflammatory properties of new substances [20]. The chemical structure of riparins I, II, III and IV is shown in Figure 1.

**Figure 2:**
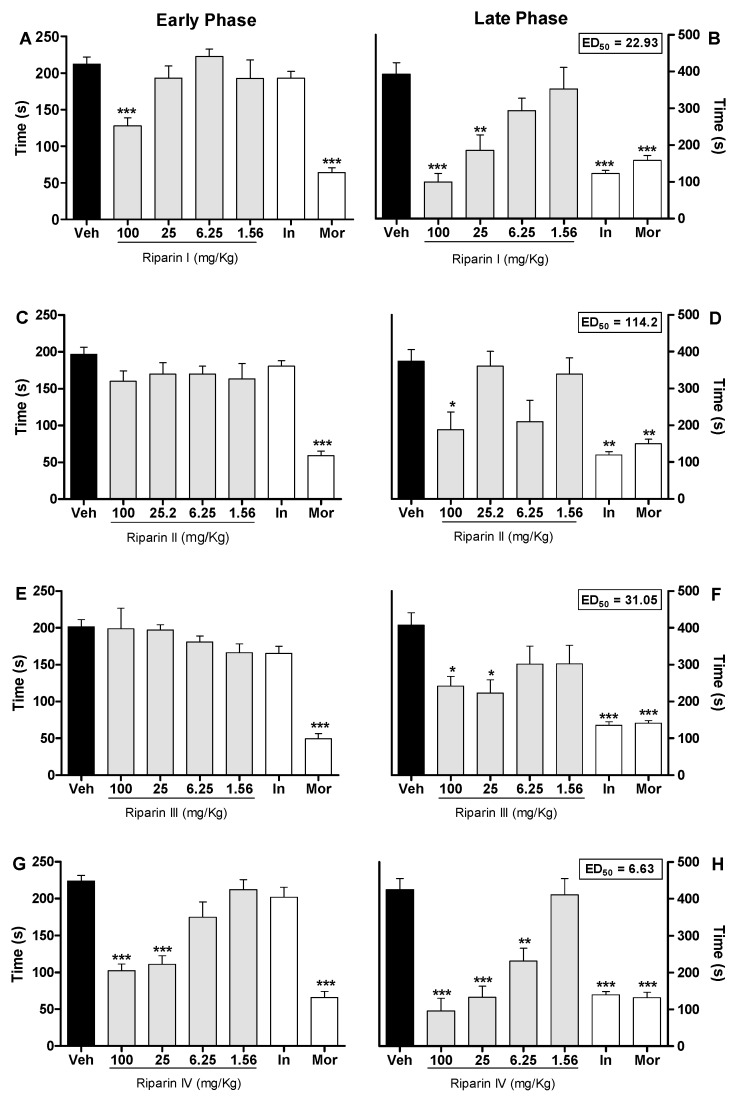
Antinociceptive effects of riparins I, II, III and IV on formalin test. Panels (**A**,**C**,**E**,**G**) represent the effects of riparins I–IV, respectively, on the early phase of formalin-induced nociception in mice. Panels (**B**,**D**,**F**,**H**) represent the effects of riparins on the late phase of formalin test. ED_50_ values for riparins were calculated using the percent of nociception reversion at late phase of the formalin test. For the dose-response analysis, the effects of increasing doses of riparins (1.56 to 100 mg/kg) were tested. Mice were treated with riparins I–IV or vehicle (Veh; Tween 80 5%; control group) by intraperitoneal route 40 min before formalin (injected at time zero). Indomethacin (In; 5 mg/kg) and morphine (Mor; 5 mg/kg) were used as reference drugs. All data are reported as means ± SEM; *n* = 6 mice per group. * Significantly different from control group (*p* < 0.05); ** Significantly different from control group (*p* < 0.01); *** significantly different from control group (*p* < 0.001) as determined by ANOVA followed by Tukey’s test.

Injection of formalin in control animals induced a biphasic flinching response, with the early phase ranging from 0 to 10 min (Figure 2A,C,E,G) and the late phase from 10 to 30 min (Figure 2B,D,F,H) after the injection. Intraperitoneal administration of riparin I (25 and 100 mg/kg; Figure 2A,B) 40 min before the injection of formalin caused antinociceptive effects in both the early and late phases of this test (*p* < 0.001). Treatment with riparin II (100 mg/kg; Figure 2C,D) and III (25 and 100 mg/kg; Figure 2E,F), 40 min before the injection of formalin, caused antinociceptive effect in the late phase of this test (*p* < 0.01).

Pretreatment with riparin IV (6.25, 25 and 100 mg/Kg; Figure 2G,H) caused antinociceptive effect in both the early and late phases of the formalin test (*p* < 0.001). The pretreatment with indomethacin (5 mg/kg, ip) inhibited the late phase, while the pretreatment with morphine (5 mg/kg, ip), a gold standard opioid, inhibited both the early and late phases of the formalin test (Figure 2). Riparins I, II and III exhibited ED_50_ values of 22.93, 114.2 and 31.05 mg/kg, respectively, to reverse the late phase of the formalin test. The antinociceptive effect of riparin IV, with ED_50_ value of 6.63 mg/kg, was three, seventeen and four folds more potent that I, II and III, respectively. The formalin test is a model of pain with two distinctive phases that may indicate different types of pain. The early phase, named nociceptive, is a result of direct stimulation of nociceptors; the late phase, named inflammatory, is caused by local inflammation with a release of inflammatory and hyperalgesic mediators [20,21].

**Figure 3:**
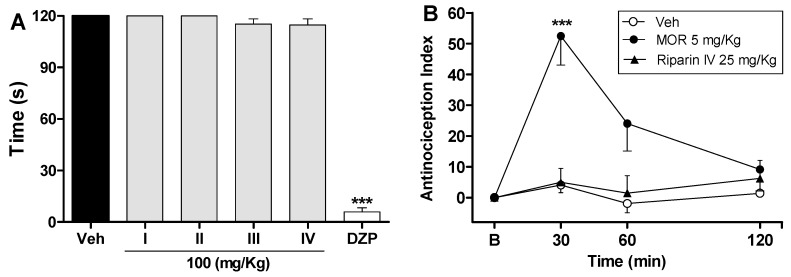
Effects of riparins on rota-rod and tail flick tests in mice. Panel (**A**) shows the effects of riparins I, II, III and IV (100 mg/kg) on motor function in mice. Bar graphs representing the run time on the rota-rod, 40 min after the intraperitoneal administration of riparins I–IV (100 mg/kg), vehicle (Veh; Tween 80 5%; control group) or diazepam (DZP; 10 mg/kg, reference drug); Panel (**B**) shows tail flick test data represented as Antinociception Index. The thermal nociceptive threshold was evaluated before (B) and up to 120 min following intraperitoneal administration of riparin IV (25 mg/kg), vehicle (Veh: Tween 80.5%; control group) or morphine (Mor: 5 mg/kg; reference drug). All data are reported as means ± SEM; *n* = 6 mice per group. *** Significantly different from control group (*p* < 0.001) as determined by ANOVA followed by Tukey’s test (Panel A) or Two-way ANOVA followed by Bonferroni’s test (B).

The antinociceptive effects of riparins were more consistent in the late phase of the formalin test, suggesting that it may involve an anti-inflammatory action. Moreover, relaxing or motor deficit effects were discarded, since administration of riparins at antinociceptive doses did not affect the motor performance in mice, as assessed by the rotarod test (Figure 3A). As expected, the central nervous system depressant diazepam (10 mg/kg ip) reduced the duration that mice were able to remain on the rota rod after 30 min of treatment with this standard drug (Figure 3A; *p* < 0.001). These results corroborate the antinociceptive effect of riparins, as suggested by the formalin test.

The preclinical stages of the drug discovery process aims to identify molecules which possess suitable characteristics to be acceptable drugs. During the lead discovery phase molecules are screened in cell-based assays and in animal models of disease to characterize both the efficacy/potency of the compound and its likely safety profile [22]. Treatment with riparins caused antinociceptive effect on the formalin test, with the following potency order: riparin IV > riparin I > riparin III > riparin II. Considering the greater potency antinociceptive displayed by riparin IV, this substance was next submitted to further investigation. Considering the inhibitory property of riparin IV on the second phase of formalin, it is possible that its antinociceptive activity is due, at least in part, to an anti-inflammatory action. Corroborating this idea, the treatment with the maximum effective dose (25 mg/Kg) of riparin IV did not prevent the nociception in the tail-flick test, a well-stablished assay to central analgesics. The reference drug morphine (5 mg/kg ip) caused a significant increase in the latency response (Figure 3B; *p* < 0.001). The thermal model of the tail flick test is considered a spinal reflex, but could also involve higher neural structures, and therefore this method identifies mainly the activity of centrally acting analgesics [23,24]. The fact that riparin IV did not induce antinociceptive effect in the tail flick test suggests that this substance does not block the neural transmission of pain, like morphine does, and may induce a peripheral analgesia.

**Figure 4:**
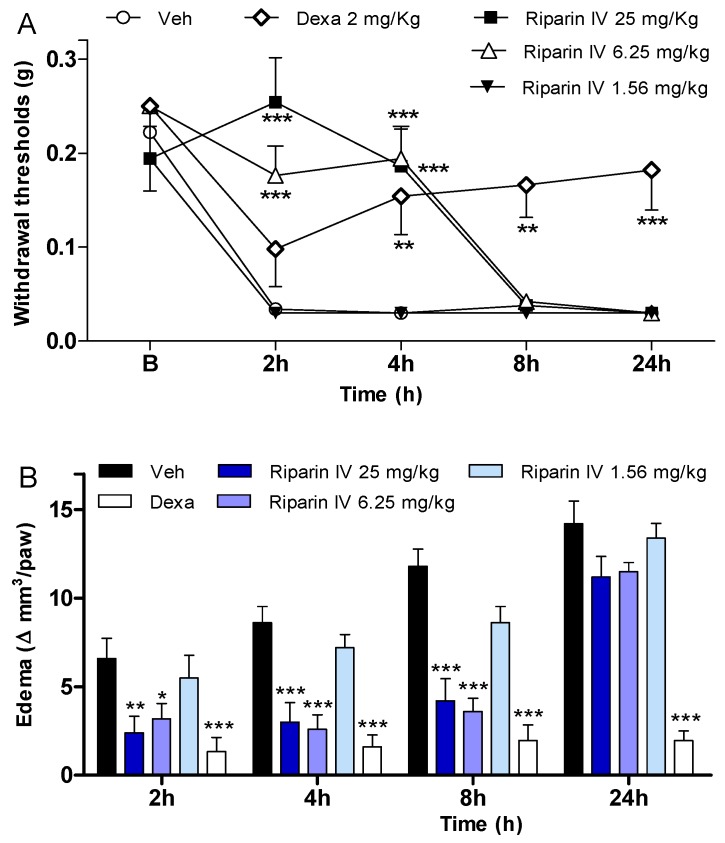
Anti-inflammatory effects of riparin IV on complete Freund’s adjuvant (CFA)-induced paw inflammation. Mice were treated with riparin IV (1.56–25 mg/kg), dexamethasone (Dexa; 2 mg/kg; reference drug), or vehicle (Veh: Tween 80 5%; control group) by ip route 40 min before the intraplantar injection of CFA (20 μL/paw). (**A**) Inflammatory hyperalgesia measured at 2, 4, 8 and 24 h after the CFA stimulus. B (basal) represents the average threshold of the animals before surgery. The mechanical nociceptive threshold (axis of ordinates) is represented as the filament weight (g) at which the animal responds in 50% of presentations; (**B**) Paw edema measured at 2, 4, 8 and 24 h after the CFA injection, represented as variation of paw volume in mm^3^. Data are expressed as means ± SEM; *n* = 6 mice per group. * Significantly different from the control group (p < 0.05); ** Significantly different from the control group (*p* < 0.01); *** Significantly different from the control group (*p* < 0.001). Two-way ANOVA followed by Bonferroni’s test.

In an attempt to verify if the antinociception induced by riparin IV is associated with anti-inflammatory properties, possible anti-inflammatory actions of this substance were investigated using the CFA-induced paw inflammation model, which is used to screen for new anti-inflammatory drugs. CFA, which consists of heat-killed mycobacteria suspended in a mineral oil vehicle, produces a chronic inflammatory condition when injected into rodents. It is well recognized that the injection of CFA produces inflammation initiated by a local release of mediators, such as cytokines and prostanoids, which are involved in the inflammatory signs, such as edema, hyperalgesia and vasodilation [25,26,27,28,29]. Therefore, the present study evaluated the effects of riparin IV on inflammatory hyperalgesia, paw edema and local levels of prostaglandins and cytokines induced by CFA. A single intraperitoneal administration of riparin IV (6.25 and 25 mg/kg), 40 min before CFA, significantly (*p* < 0.001) reduced inflammatory hyperalgesia at 2 and 4h after stimulus (Figure 4A). It is important to mention that pretreatment with riparin IV did not alter the baseline nociceptive threshold of the animals (data not shown). The administration of CFA into the right hind paw induced gradually heightened edema until reaching its peak 24 h after injection. The administration of riparin IV (6.25 and 25 mg/Kg ip) 40 min before CFA reduced significantly (*p* < 0.001) the paw edema 2, 4 and 8 h post-stimulus (Figure 4B). Maximum effective dose of riparin IV against CFA-induced paw edema was 6.25 mg/Kg. The results obtained with control groups support the observed anti-inflammatory effects for riparin IV as vehicle treatment (Tween 80 5% in saline) yielded no activity, however the standard drug dexamethasone (2 mg/kg), inhibited CFA-induced hyperalgesia (*p* < 0.01) and edema (*p* < 0.001) until 24 h after stimulus (Figure 4).

**Figure 5:**
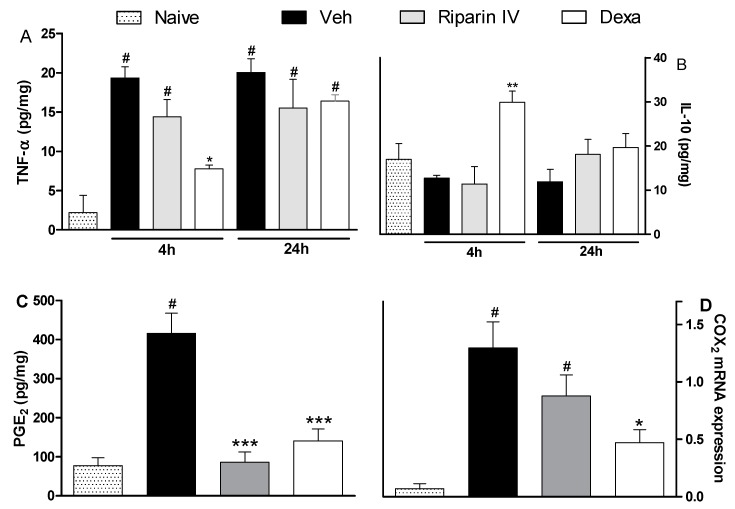
Effects of riparin IV on paw inflammatory mediators levels during CFA-induced inflammation. Mice were treated ip with riparin IV (6.25 mg/kg), dexamethasone (Dexa; 2 mg/kg; reference drug), or vehicle (Veh: Tween 80 5%; control group) 40 min before the intraplantar injection of CFA (20 μL/paw). The naïve group consists of mice that did not receive any experimental manipulation. Panels show the paw levels of (**A**) tumor necrosis factor-α (TNF-α), (**B**)interleukin-10 (IL-10) or (**C**) prostaglandin E_2_ (PGE_2_), determined in skin tissues samples 3 h after the CFA injection. The results are expressed as picograms of cytokine per milligram of protein. Panel D shows the paw levels of COX-2 mRNA measured by qRT-PCR 3 h after the CFA injection. All data are reported as means ± SEM; *n* = 6 mice per group. * Significantly different from control group (*p* < 0.05); ** Significantly different from control group (*p* < 0.01); *** Significantly different from control group (*p* < 0.001); ^#^ Significantly different from the naive group (*p* < 0.05) as determined by ANOVA followed by Tukey’s test.

The antinociceptive effect of riparin IV on the CFA model had a greater efficacy than that of the gold standard dexamethasone, however, it was short-lasting. This effect profile may suggest that these substances are acting through different mechanisms of action. Data obtained by ELISA analyses corroborates this idea. Unlike dexamethasone, riparin IV did not modulate the production of inflammatory cytokines during CFA-induced paw inflammation. The paw levels of TNF-α and IL-10 were not modulated by riparin IV at 6.25 mg/kg, the anti-edematogenic maximum effective dose (Figure 5A,B).

As expected, the pretreatment with dexamethasone (2 mg/kg) reduced TNF-α (*p* < 0.05) and enhanced IL-10 (*p* < 0.01) paw levels on CFA-induced inflammation (Figure 5; *p* < 0.05). Inflammatory cytokines play an essential role in the development of inflammatory signs and symptoms, and its release is followed by the release of anti-inflammatory cytokines, which limit the deleterious consequences of prolonged inflammatory reaction [30]. The present study indicates that riparin IV induces anti-inflammatory effect independent of cytokine modulation. 

Based on the relevant anti-inflammatory action of non-steroidal anti-inflammatory drugs and its cyclooxygenase inhibitory effect, it is accepted that prostaglandins are important contributors of inflammatory response. Aiming to evaluate the contribution of the prostanoid signaling to the anti-inflammatory effects of riparin IV, the PGE_2_ levels and COX-2 expression were next evaluated. Mice pretreated with riparin IV (6.25 mg/kg, i.p.) or dexamethasone (2 mg/kg) had a significant (*p* < 0.001) decrease in PGE_2_ levels in the paw after CFA injection (Figure 5C). As shown by qRT-PCR analysis, riparin IV (6.25 mg/kg, i.p.) was not able to reduce the COX-2 mRNA expression in the paw during inflammation (Figure 5D). On the other hand, a reduction of COX-2 mRNA expression was observed in the paws of dexamethasone-treated mice (*p* < 0.05). Riparin IV decreased the PGE_2_ levels, but not COX-2 mRNA expression, in the paw after the CFA stimuli. Therefore, it is possible that riparin IV acts by preventing the enzymatic production of final mediators of inflammation. Inflammation causes the induction of COX-2, leading to the release of prostanoids, which contribute to the development of peripheral sensitization through phosphorylation of ion channels in nociceptor terminals, increasing excitability and reducing the pain threshold [31,32]. While the sensitization of nociceptors does not by itself provoke overt pain, it is common to all types of inflammatory pain and is associated with chronic pain [33]. Considering that the antinociceptive effect riparin IV is associated with a decrease of PGE_2_ levels, its antinociceptive effect could be a reflex of a reduced sensitization of the nociceptors. 

**Figure 6:**
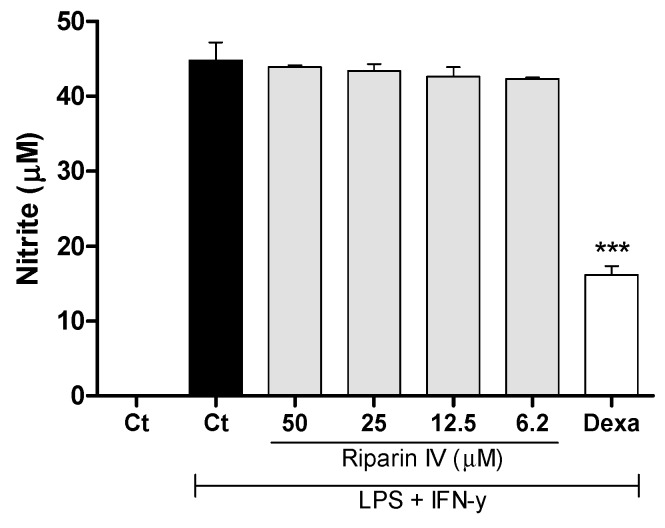
Effect of riparin IV on nitric oxide production in J774 macrophages. Concentrations of nitrite were determined in J774 macrophages treated with vehicle (Tween 80 5%, Ct, control group), riparin IV (6.2–50 μM) or dexamethasone (Dexa; 40 µM) in the presence of LPS (500 ng/mL) + IFN-γ (5 ng/mL). Cell-free supernatants were collected for nitrite quantification by the Griess method. Values represent the means ± SEM of three determinations obtained in one of three experiments performed. *** Significantly different from the vehicle treated cultures (*p* < 0.001). ANOVA followed by Tukey´s multiple comparison test.

Aiming to understand the mechanism of action of riparin IV, a set of in vitro assays was performed. Macrophages play a central role in the immune regulation having functions such as antigen presentation, phagocytosis, immunomodulation and production of inflammatory mediators such as nitric oxide (NO) [34]. NO is a crucial molecule in the regulation of vascular tone, acute and chronic inflammation and host defense mechanisms [35]. Activated macrophages are capable of releasing high levels of NO in certain types of inflammation, and NO has been implicated as a pro-inflammatory agent [36]. Therefore, in the present study a possible suppressive effect of riparin IV in macrophages was evaluated. Firstly, the cytotoxic effect of riparin IV on cell viability was determined using a colorimetric Alamar Blue assay after 24 h of treatment. Riparin IV at a concentration of 200 µM or lower did not induce cytotoxic effects in J774 macrophages (data not shown in figures). The suppressive effects of riparin IV in macrophages were investigated through the quantification of nitric oxide and cytokine production by stimulated macrophages. As shown in Figure 6, the treatment with riparin IV did not reduced the production of nitrite in J774 macrophages stimulated with LPS and IFN-γ. Dexamethasone (40 µM), the gold standard, was able to reduce nitrite production (*p* < 0.001).

**Figure 7:**
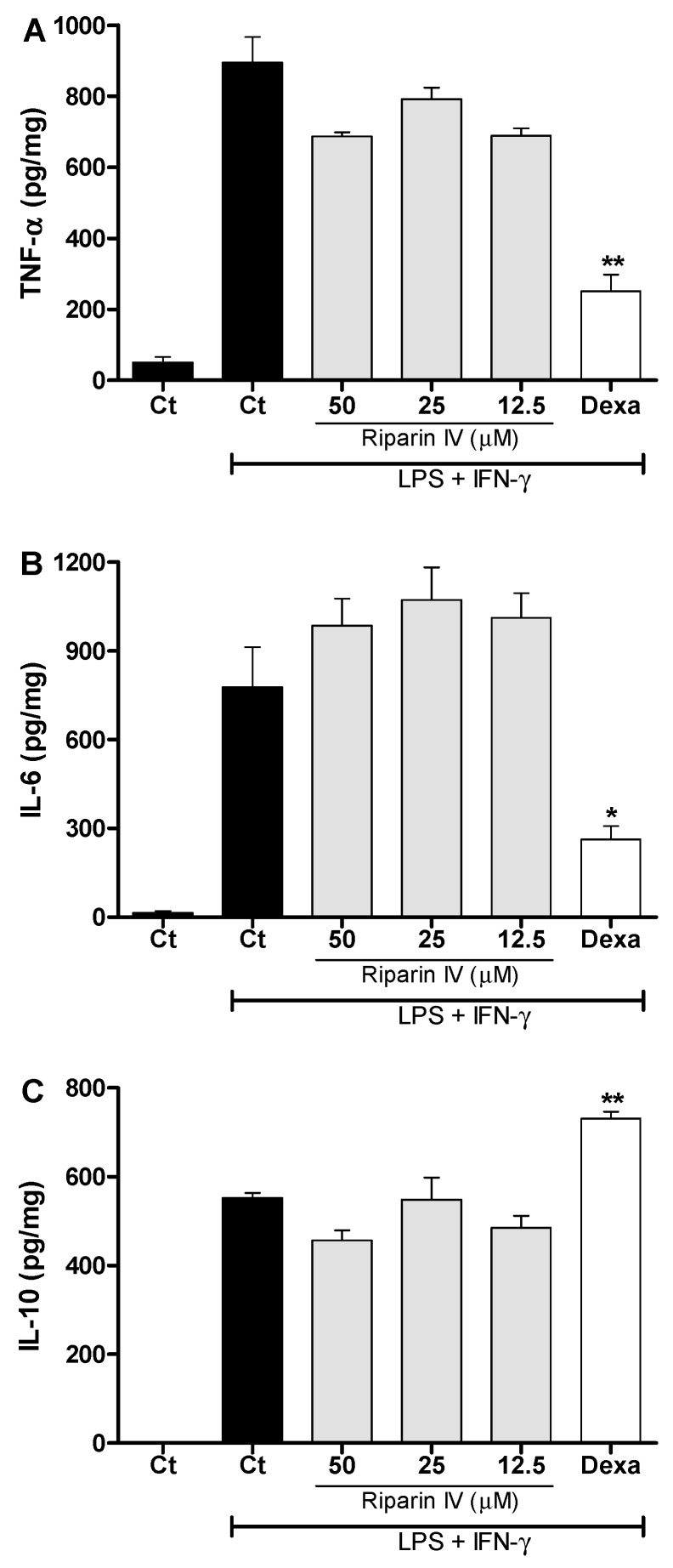
Effect of riparin IV on cytokine production by activated J774 macrophages. Concentrations of TNF-α (**A**), IL-6 (**B**) and IL-10 (**C**) were determined in cultures of J774 macrophages treated with vehicle (Tween 80 5%, Ct, control group), riparin IV (6.2–50 μM) or dexamethasone (Dexa; 40 µM) in the presence of LPS (500 ng/mL) plus IFN-γ (5 ng/mL). Cell-free supernatants were collected for cytokines measurement by ELISA. Values represent the means ± SEM of four determinations obtained in one of three experiments performed. * Significantly different from the vehicle treated cultures stimulated with LPS + IFN-γ (*p* < 0.05). ** *p* < 0.05. ANOVA followed by Tukey’s multiple comparison test.

Figure 7 shows that the riparin IV (12.5–50 μM) did not modulate the production of TNF-α, IL-6 and IL-10 in J774 macrophages stimulated with LPS and IFN-γ. Under the same conditions, dexamethasone (40 μM) was able to reduce the levels of pro-inflammatory cytokines, TNF-α and IL-6 (*p* < 0.05), while enhanced the anti-inflammatory cytokine IL-10 (*p* < 0.01) in activated macrophages. These results demonstrated that inhibition of NO and pro-inflammatory cytokines by activated macrophage is not a mechanism involved in the anti-inflammatory effect of riparin IV. Since LPS upregulates the expression of Cox2 [37], however, it is possible that riparin IV also modulates the production of PGE_2_ by LPS-activated macrophages, as seen in the in vivo CFA model.

In conclusion, to the best of our knowledge, this is the first demonstration of the pharmacological properties of riparin IV. Overall, the present results suggest that the antinociceptive and anti-inflammatory effects of riparin IV are related to its notable ability to inhibit the production of PGE_2_ deprived of immunosuppressive action. These results highlight the need of further investigations of the possible use of riparin IV as a prototype compound for development of new drugs to treat inflammatory pain.

## 3. Materials and Methods

### 3.1. Animals

Experiments were performed on male Swiss Webster mice obtained from the Animal Facilities at the Centro de Pesquisas Gonçalo Moniz (Salvador, Brazil). Animals (22–28 g) were housed in temperature-controlled rooms (22–25 °C), under a 12:12 h light-dark cycle, with access to water and food ad libitum until experimental initiation. All behavioral tests were performed between 8:00 a.m. and 5:00 p.m., and animals were only used once. Animal care and handling procedures were in accordance with the National Institutes of Health guide for the care and use of Laboratory animals (NIH, 8023) and the Institutional Animal Care and Use Committee FIOCRUZ (L-IGM-015/2013). Every effort was made to minimize the number of animals used and any discomfort. Behavioral tests were performed without knowing to which experimental group each mouse belonged. Results shown are from two independent experiments performed.

### 3.2. Compounds, Dilutions and Administration

In the present study riparins was synthesized from substituted synthetic benzoic acids using methodology previously described by Barbosa-Filho and co-workers [19]. Riparin IV (chemical name *N*-[8′-(4′-methoxyphenylethyl)]-3,4,5-trimethoxybenzoylamide), is a synthetic amide modeled on the natural riparins isolated from *Aniba riparina.* Dexamethasone, indomethacin, complete Freund’s adjuvant (CFA), lipopolysaccharide (LPS) and interferon-γ (IFN-γ) were obtained from Sigma Chemical Company (St. Louis, MO, USA). Diazepam and morphine were obtained from Cristália (Itapira, SP, Brazil). Riparins were dissolved in 5% Tween 80 in distilled water. Indomethacin was dissolved in Tris HCl 0.1 M pH 8.0 plus distilled water. Dexamethasone was dissolved in ethanol 10% in distilled water, and remaining substances were dissolved directly in distilled water. Test compounds were administrated by intraperitoneal (ip) route 40 min before testing, and the control group only received vehicle.

### 3.3. Formalin Test

Mice were placed in an open Plexiglas observation chamber for 30 min to acclimate to their surroundings, and then removed for formalin administration. Mice were gently restrained while 20 µL of 2.5% formalin (1:100 dilution of stock formalin solution, 37% formaldehyde in 0.9% saline) was administered subcutaneously (sc) to the dorsum of the hind paw using a 30 gauge needle. Following injection, mice were returned to the observation chamber for a 30 min observation period. A mirror was placed behind the chamber to enable unhindered observation of the formalin-injected paw. Mice were observed from 0 to 10 min (early phase) and from 10 to 30 min (late phase), and a nociception score was determined for each period by counting the time that the animal spent licking, biting or elevating the injected limb during the observation time [38]. Mice were treated with riparins (1.56–100 mg/Kg), vehicle (5% Tween 80 in distilled water; control group), indomethacin (5 mg/kg; reference drug) or morphine (5 mg/kg; reference drug), by ip route 40 min before formalin.

### 3.4. Tail Flick Test

The warm-water tail withdrawal test in mice was conducted as described previously [39]. The day before the experiment, each animal was habituated to the restraint cylinder for 20 min/day for 5 consecutive days. On the day of the experiment, mice were placed in the restraint cylinder and the tail tip (2 cm) was submersion in a water bath at 48 ± 0.5 °C. The latency of the tail withdrawal reflex was measured in seconds. Each submersion was terminated after 12 s to minimize potential skin damage. Tail flick latency was measured before (baseline) and after treatments.

### 3.5. Motor Function Assay

To evaluate the possible non-specific muscle-relaxant or sedative effects of riparins, mice were submitted to the rota-rod test, as previously described [39]. The rota-rod apparatus (Insight, Ribeirão Preto, Brazil) consisted of a bar with a diameter of 3 cm, subdivided into five compartments. The bar rotated at a constant speed of 8 revolutions per minute. The animals were selected 24 h previously by eliminating those mice that did not remain on the bar for two consecutive periods of 120 s. Animals were treated with riparins (100 mg/Kg), diazepam (10 mg/Kg, reference drug) or vehicle (5% Tween 80 in distilled water; control group) and 40 min afterward were placed on a rotating rod. The resistance to falling was measured up to 120 s. The results are expressed as the average time (s) the animals remained on the rota-rod in each group.

### 3.6. Inflammatory Model

Mice were lightly anesthetized with halothane and received 20 µL of complete Freund’s adjuvant (CFA 1 mg/mL of heat killed *Mycobacterium tuberculosis* in 85% paraffin oil and 15% mannide monoleate) in the plantar region of the right hind paw, according to a previously reported method [11]. Inflammatory hyperalgesia, edema, local cytokines and prostaglandin-E_2_ levels, and expression of COX-2 were measured by von Frey filaments, plesthismometer, ELISA and PCR, respectively, as described below. Mice were injected with riparin IV (1.56–100 mg/kg), vehicle (5% Tween 80 in distilled water; control group) or dexamethasone (2 mg/Kg, reference drug) by ip route 40 min before CFA.

### 3.7. Inflammatory Hyperalgesia Evaluation

The threshold to mechanical stimulation was measured with von Frey filaments (Stoelting, Chicago, IL, USA). In a quiet room, mice were placed in acrylic cages (12 × 10 × 17 cm) with wire grid floors 30 min before the beginning of the test. This consisted of evoking a hind paw flexion reflex with one of a series of filaments with logarithmically incremental stiffness (0.0045–28.84 g). A positive response was characterized by the removal of the paw followed by clear flinching movements. A tilted mirror placed under the grid provided a clear view of the hind paws of the mice. An up-down method was used to record the threshold, which was represented as the filament weight (g) in which the animal responds in 50% of presentations [40].

### 3.8. Plesthismometer Test

The volume of each mouse paw was measured (mm^3^) with a plesthismometer (Ugo Basile, Comerio, Italy) before (Vo) and after (VT) the CFA injection, as described previously [11]. The amount of paw swelling was determined for each mouse and data were represented as paw volume variation (Δ, mm^3^).

### 3.9. Cytokine Measurement by ELISA

The paw levels of cytokines were determined as previously described [11]. Treatments were performed 40 min before the CFA injection. Skin tissues were removed from the paws 4 and 24 h after CFA, in mice terminally anesthetized with halothane from each experimental group. Tissue proteins were extracted from 100 mg tissue/mL phosphate buffered saline (PBS) to which 0.4 M NaCl, 0.05% Tween 20 and protease inhibitors (0.1 mM PMSF, 0.1 mM benzethonium chloride, 10 mM EDTA, and 20 KI aprotinin A/100 mL) were added (Sigma Chemical Company, St. Louis, MO, USA). The samples were centrifuged for 10 min at 3000 g and the supernatant was frozen at −70 °C for later quantification. Tumor necrosis factor α (TNF-α) and interleukin-10 (IL-10) levels were estimated using commercially available immunoassay ELISA kits for mice (R&D System, Minneapolis, MN, USA), according to the manufacturer’s instructions. The results are expressed as picograms of cytokine per milligram of protein.

### 3.10. Measurement of PGE_2_ in Paw Skin

The plantar tissues were collected 3 h after intraplantar injection of CFA (20 µL/paw). The paws were injected with indomethacin (50 μg/paw) 10 min before tissue retrieval to block PGE_2_ production during tissue processing. The PGE_2_ levels were determined by radioimmunoassay, as previously described [41]. Briefly, the plantar tissue samples were homogenized in a mixture of 3.0 mL of extraction solvent (isopropanol/ethyl acetate/0.1 N HCl, 3:3:1) and 3.0 mL of distilled water contained 20 μg/mL of indomethacin. Homogenates were centrifuged at 1500× *g* for 10 min at 4 °C. The organic phase was aspirated and evaporated to dryness in a centrifugal evaporator. The pellet was reconstituted in 500 μL of 0.1 M phosphate buffer (pH 7.4) containing 0.8% sodium azide and 0.1% gelatin. Concentration of PGE_2_ in these samples was then measured by RIA by using a commercially available kit. The results are expressed as picograms of PGE_2_ per milligram of protein.

### 3.11. Real-Time PCR

The transcription of cyclooxygenase-2 (COX-2) gene was evaluated by real-time quantitative polymerase chain reaction (qRT-PCR) in mice sacrificed 3h after the CFA injection. Total RNA was isolated from the paw tissue with TRIzol reagent (Invitrogen Corp., Carlsbad, CA, USA), and the concentration was determined by photometric measurement. A High Capacity cDNA Reverse Transcription Kit (Applied Biosystems, Foster City, CA, USA) was used to synthesize cDNA from 1 µg of RNA following the manufacturer’s recommendations. The qRT-PCR assay was performed to detect the expression levels of COX-2 gene. Amplification mixtures for qRT-PCR contained 20 ng template cDNA, 10 µL Taqman Master Mix (Applied Biosystems) and probes in a final volume of 20 µL. All reactions were run in duplicate on an ABI7500 Sequence Detection System (Applied Biosystems) under standard thermal cycling conditions. Experiments with coefficients of variation greater than 5% were excluded. A no-template control (NTC) and no-reverse transcription controls (No–RT) were also included. The results are presented as the fold-increase of COX-2 mRNA, with the target internal control GADPH using the cycle threshold method.

### 3.12. Cytotoxicity to Mammalian Cells

To determine the cytotoxicity of riparin IV, the murine macrophage-like cell line J774 was plated into 96-well plates at a cell density of 2 × 10^5^ cells/well in Dulbecco’s modified Eagle medium (DMEM; Life Technologies, GIBCO-BRL, Gaithersburg, MD, USA) supplemented with 10% fetal bovine serum (FBS; GIBCO), and 50 µg/mL of gentamycin (Novafarma, Anápolis, GO, Brazil) and incubated for 2 h at 37 °C and 5% CO_2_. After that time, riparin IV were added at four concentrations ranging from 3.1 to 200 µM in triplicate and incubated for 24 h. Twenty µL/well of Alamar Blue (Invitrogen) was added to the plates during 12 h. Colorimetric readings were performed at 570 and 600 nm. Gentian violet (Synth, São Paulo, Brazil) at 10 μM was used as positive control. Three independent experiments were performed.

### 3.13. Assessment of Cytokine and Nitric Oxide Production by Macrophages

For the evaluation of cytokine and nitric oxide production, J774 cells were seeded in 96-well tissue culture plates at 2 × 10^5^ cells/well in DMEM medium supplemented with 10% of FBS and 50 µg/mL of gentamycin for 2 h at 37 °C and 5% CO_2_. Cells were then stimulated with LPS (500 ng/mL, Sigma Chemical Co.) and IFN-γ (5 ng/mL; Sigma) in the presence of riparin IV, vehicle or dexamethasone at different concentrations, and incubated at 37 °C. Cell-free supernatants were collected 4 h (for TNF-α measurement) and 24 h (for IL-10, IL-6 and nitrite quantification) and kept at −80 °C. Cytokine concentrations in supernatants from J774 cultures were determined by enzyme-linked immunosorbent assay (ELISA), using the DuoSet kit from R&D Systems, according to the manufacturer’s instructions. Quantification of nitric oxide was estimated by assessing the nitrite concentrations using the Griess method [42]. 

### 3.14. Statistical Analysis

Data are presented as means ± standard error of the means (SEM) of measurements made on 6–9 animals in each group. Comparisons between three or more treatments were made using one-way ANOVA with Tukey’s post-hoc test, or for repeated measures, two-way ANOVA with Bonferroni’s post-hoc test, as appropriate. All data were analyzed using Prism 5 Computer Software (GraphPad, San Diego, CA, USA). Statistical differences were considered to be significant at *p* < 0.05. ED_50_ values were calculated using the percent of nociception reversion at late phase of the formalin test (Prism 5).
